# Overexpression of IL-8 augments the susceptibility to a hyperinflammatory phenotype in pediatric acute respiratory distress syndrome and correlates with adverse outcomes: a retrospective two-center study conducted in northwest China

**DOI:** 10.3389/fped.2026.1626327

**Published:** 2026-02-03

**Authors:** Yi Wang, Weikai Wang, Zhe Lv, Haitong Wu, Hua Zhang, Ying Wang, Yong Zhou, Zhangyan Guo, Jingmei Li, Le Ma, Dan Yao, Taining Zhang, Yanqiang Du, Li Liu

**Affiliations:** 1 Department of Neonatology, The First Affiliated Hospital of Xi’an Jiaotong University, Xi’an, Shaanxi, China; 2 Pediatric Intensive Care Unit, The Affiliated Children’s Hospital of Xi’an Jiaotong University, Xi’an, Shaanxi, China; 3 Pediatric Intensive Care Unit, Gansu Provincial Maternity and Child-care Hospital, Gansu Provincial Central Hospital, Lanzhou, Gansu, China; 4 Department of Medicine, Xi’an Jiaotong University Health Science Center, Xi’an, Shaanxi, China

**Keywords:** acute respiratory distress syndrome, biomarkers, interleukin-8, pediatric, receptor for advanced glycation endproducts

## Abstract

**Background:**

The prognosis of acute respiratory distress syndrome (ARDS) varies with inflammatory responses. ARDS patients with a hyperinflammatory phenotype usually have worse alveolar epithelial injury and vascular endothelial injury than those carrying a hypoinflammatory phenotype. Activated neutrophils recruited and migrated in the lung tissue are responsible for stimulating the progression of ARDS. Interleukin-8 (IL-8), as an inflammatory factor, further aggravates lung damage in ARDS.

**Methods:**

This was a retrospective study involving 135 ARDS children admitted in two pediatric hospitals in northwest China. They were either classified into mild, moderate and severe groups based on the oxygenation index (OI) or oxygenation saturation index (OSI) within 4-h invasive mechanical ventilation on admission, or the survival and non-survival groups based on the 28-day mortality. Demographic and clinical data were analyzed. Risk factors for the prognosis of PARDS were identified by logistic regression. The correlation of IL-8 level with the identified risk factors was analyzed. Prognostic potential of IL-8 was determined by plotting the receiver operating characteristic (ROC) curves.

**Results:**

IL-8, RAGE, Ang-2, ICAM-1 and SP-D were independent risk factors for the mortality of PARDS. They were significantly higher in the non-survival group than the survival group, showing a potential in predicting mortality in PARDS, especially in the combination (*P* < 0.05). IL-8 was positively correlated with RAGE, Ang-2, ICAM-1 and SP-D in children with ARDS (*P* < 0.05).

**Conclusion:**

IL-8 is overexpressed in children with ARDS, showing a prognostic potential particularly in combination with RAGE, Ang-2, ICAM-1 and SP-D in PARDS.

## 1 Introduction

The acute respiratory distress syndrome (ARDS) is a life-threatening condition caused by pathological alterations in the alveolar epithelium and capillary epithelium. It is a heterogeneous syndrome mainly manifested as pulmonary edema, increased capillary permeability and refractory hypoxemia (1–3). Inflammatory factors exert a pivotal role in the lung diseases (4). The severity of inflammatory response predicts the prognosis of ARDS. Biomarkers associated with inflammation response have been proposed to aid in the diagnosis, risk stratification, and classification of ARDS (5). Inflammatory mediators (6, 7), damage-related molecular patterns (8), and endothelial (9–11) and epithelial injury markers are closely linked with adult ARDS (11–13).

Disease heterogeneity is a well concerned issue especially in pediatric intensive care. PARDS is a highly heterogenous condition, presenting varied manifestations with age, causes (pneumonia, sepsis, or trauma), pre-existing comorbid conditions, and baseline immunological state. Classified by the inflammatory subtype, ARDS can be either hyperinflammatory or hypoinflammatory, showing distinct manifestations and outcomes (4, 14). PARDS affects 3% of PICU patients, and accounts for 30% of mortality in those with severe hypoxaemia (15). Specifically, pediatric patients exhibiting high levels of inflammatory responses demonstrate a significantly elevated mortality rate of ARDS.

In the present study, we analyzed clinical data and inflammatory factors in children with ARDS, who were stratified based on the disease severity. Moreover, we specifically explored the role of IL-8 in the hyperinflammatory phenotype of PARDS, aiming to provide references for clinical management of PARDS.

## 2 Methods

### 2.1 Study design

This was a retrospective study involving 135 children (28 days to 18 years of age) diagnosed with ARDS in two specialized pediatric hospitals in northwest China, namely the Children's Hospital Affiliated to Xi'an Jiaotong University and Gansu Provincial Maternity and Child Health Hospital from February 2021 to November 2023. Retrospective data study can be exempted from the informed consent.

The study was approved by the Ethics Committee of the Affiliated Children's Hospital of Xi'an Jiaotong University (No.20240022) and Gansu Provincial Maternity and Child Health Hospital, Gansu Provincial Central Hospital (No.20230011). Written informed consent was provided by guardians. This study adhered to principles of the Declaration of Helsinki.

### 2.2 Participants

According to the Second Pediatric Acute Lung Injury Consensus Conference (PALICC-2) definition of ARDS, a diagnosis of ARDS was established as follows: (I) hypoxemia occurs within 7 days of a clinical insult; (II) new opacities (unilateral or bilateral) consistent with acute pulmonary parenchymal disease that are not explained by atelectasis or pleural effusion; (III) not fully explained by cardiac failure or fluid overload; (IV) hypoxemia essential for respiratory support by mechanical ventilation.

Respiratory support for non-invasively ventilated (NIV) participants was performed using a full-face mask or nasal mask in a continuous positive airway pressure or bilevel positive airway pressure ≥5 cm H_2_O, achieving the goal of the ratio of partial arterial pressure of oxygen (PaO_2_) to fractional concentration of inspired oxygen (FiO_2_) ≤ 300, or the ratio of blood oxygen saturation to FiO_2_ (SF ratio) ≤ 264. Mechanical ventilation in invasively ventilated participants achieved the following goal: oxygenation index (OI) ≥ 4 or oxygenation saturation index (OSI) ≥ 5.

**Figure 1:**
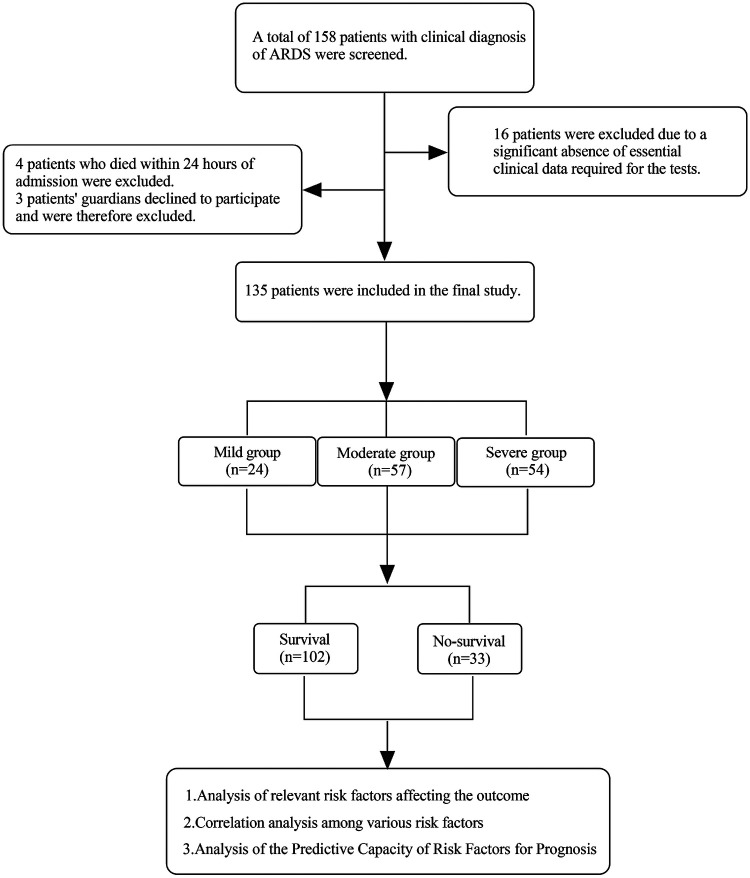
A flowchart of the study design.

A total of 158 children diagnosed with ARDS and admitted to the hospital were initially screened. Of these, 16 were excluded due to incomplete clinical data, and an additional 7 were excluded—4 who died within 24 h of admission and 3 whose legal guardians declined participation in the study. 135 eligible children with ARDS were classified into mild (4 ≤ OI < 8 or 5 ≤ OSI < 7.5), moderate (8 ≤ OI < 16 or 7.5 ≤ OSI <12.3) and severe groups (OI ≥ 16 or OSI≥12.3) based on the OI or OSI within 4-h invasive mechanical ventilation on admission. NIV patients were included in the mild group. Based on the 28-day mortality, participants were assigned into survival group (*n* = 102) and non-survival group (*n* = 33) (Figure 1).

### 2.3 Data collection

Biologically banked blood specimens of each participant were subjected to measurements of angiopoietin-2 (Ang-2), von Willebrand factor (vWF), D-dimer, blood cell analysis parameters, intracellular adhesion molecule-1 (ICAM-1), receptor for advanced glycation endproducts (RAGE), surfactant protein D (SP-D), and inflammatory factors in duplicates.

Demographic data, etiological factors, cause of ARDS, use of extracorporeal membrane oxygenation (ECMO), use of mechanical ventilation, the Lung Injury Score (LIS), complications, treatment process and 28-day mortality were retrospectively collected as well.

### 2.4 Statistical analysis

Statistical analysis was performed using SPSS 21.0. Continuous variables in a normal distribution were expressed as mean ± standard deviation (SD), and compared between groups by the independent samples *t*-test; otherwise, they were expressed as median and interquartile range (IQR), and compared by the Mann–Whitney *U*-test. Categorical variables were compared by the Chi-square test. Risk factors were identified by binary logistic regression, and correlation of two variables was determined by a linear regression. The receiver operating characteristic (ROC) curves were plotted to examine the diagnostic potential. *P* < 0.05 suggested a significant difference.

## 3 Results

### 3.1 Baseline characteristics of children with ARDS

Totally 135 children with ARDS were assigned into the mild group (*n* = 24), moderate group (*n* = 57) and severe group (*n* = 54). No significant differences were detected in the gender and age among the three groups (*P* > 0.05). There were no statistically significant differences among the three patient groups with respect to the distribution of etiological factors in infection-associated pneumonia [10[41,67] vs. 32[56.14] vs. 38[70.37]], non-pulmonary sepsis [6[25] vs. 10[17.54] vs. 7[12.95]], drowning [0 vs. 2 [3.51] vs. 3[5.56]] and pancreatitis [0 vs. 3[5.27] vs. 3[5.56]]. A significant difference was only detected in the proportion of aspiration pneumonia [8[33.33] vs. 10[17.54] vs. 3[5.56]] among the three groups (*P* < 0.05). The pathogen of viral infection in the mild group [3(12.5)] was lower than that in the moderate group [24 (42.11)] and severe group [20 (37.04)] (*P* < 0.05). However, no statistically significant difference was found between the moderate and severe groups.

Compared with those of mild group, we defected significantly higher LIS, proportions of pneumothorax, use of ECMO, mechanical ventilation, multiple organ disfunction syndrome (MODS) and adjuvant therapies (renal replacement, use of inhaled nitric oxide, neuromuscular blockade and use of corticosteroids), and 28-day mortality in moderate and severe groups (*P* < 0.05, Table 1).

**Table 1:** General clinical characteristics of the three study groups (*n* = 135).

	Mild group (*n* = 24)	Moderate group (*n* = 57)	Severe group (*n* = 54)
Male (n, %)	14 (58.33)	27 (47.37)	25 (46.30)
Age (n, %)			
≥28 days, <3 yrs	5 (20.83)	8 (14.04)	7 (12.96)
≥3 yrs, <6 yrs	8 (33.33)	16 (28.07)	17 (31.49)
≥6 yrs, < 18 yrs	11 (45.84)	33 (57.89)	30 (55.55)
Etiology (n, %)			
Infection-associated pneumonia	10 (41.67)	32 (56.14)	38 (70.37)
Aspiration pneumonia	8 (33.33)	10 (17.54)	3 (5.56)£&
Non-pulmonary sepsis	6 (25)	10 (17.54)	7 (12.95)
Drowning	0	2 (3.51)	3 (5.56)
Pancreatitis	0	3 (5.27)	3 (5.56)
Pathogen (n, %)			
Bacteria	8 (33.33)	11 (19.29)	21 (38.88)
Virus	3 (12.5)	24 (42.11)	20 (37.04)£
Mycosis	2 (8.33)	7 (12.28)	5 (9.26)
Others	1 (4.17)	5 (8.77)	2 (3.7)
LIS (median, points)	2 (1, 2.5)	3 (2, 4.5)	5 (3.5, 7.5)£&
Pneumothorax (n, %)	1 (4.17)	6 (10.53)	18 (33.33)£&
Adjuvant therapies (n, %)			
Renal replacement	2 (8.33)	9 (15.79)	16 (26.93)£&
Use of Inhaled nitric oxide	3 (12.5)	17 (29.82)	28 (51.85)£&
Neuromuscular blockade	2 (8.33)	25 (43.86)	26 (48.15)£&
Use of corticosteroids	3 (12.5)	12 (21.05)	26 (48.15)£&
Use of ECMO (n, %)	0	8 (14.03)	19 (35.19)£&
Mechanical ventilation (n, %)	15 (62.5)	57 (100)	54 (100)£
Blood cell analysis			
Platelet count (×109/L)	167 ± 56	121 ± 34	68 ± 32£&
Leukocyte count (×109/L)	3.5 (2.32, 4.91)	4.7 (2.55, 7.54)	7.5 (3.35, 14.65)
Neutrophils (%)	57 ± 28	45 ± 32	53 ± 21
ymphocytes (%)	33 ± 12	36 ± 19	34 ± 15
Hb (g/dL)	143 ± 37	123 ± 41	76 ± 29
Inflammatory factors			
CRP (mg/L)	45 ± 23	67 ± 31	49 ± 22
PCT (mg/L)	4 ± 3	6 ± 5	6 ± 5
IL-8 (pg/mL)	21 ± 11	42 ± 8	74 ± 11£&
TNF-α (pg/mL)	45 ± 12	41 ± 9	55 ± 6
Lung injury biomark			
D-dimer (×109/L)	5.52 ± 2.67	9.56 ± 6.75	15.78 ± 7.98£&
vWF (mg/L)	321 ± 119	564 ± 267	774 ± 116£&
RAGE (pg/L)	1,354 ± 354	1,895 ± 123	2,235 ± 334£&
Ang-2 (pg/L)	43 ± 12	66 ± 21	89 ± 13£&
ICAM-1 (ng/mL)	798 ± 145	1,456 ± 321	1,973 ± 434£&
SP-D (pg/mL)	11 ± 6	18 ± 4	27 ± 11£&
Outcomes			
MODS (n, %)	2 (8.33)	13 (22.81)	21 (38.89)£&
28-day mortality	2 (8.33)	12 (21.05)	19 (35.19)£&

### 3.2 Clinical features of children with PARDS

There were significant differences in the D-dimer, vWF, platelet count, RAGE, Ang-2, ICAM-1 and SP-D levels among the three groups (*P* < 0.05, Table 1). Notably, IL-8 was an inflammatory marker showing a significant difference in children with ARDS stratified by the severity (*P* < 0.05).

### 3.3 Risk factors for the mortality of PARDS

**Figure 2:**
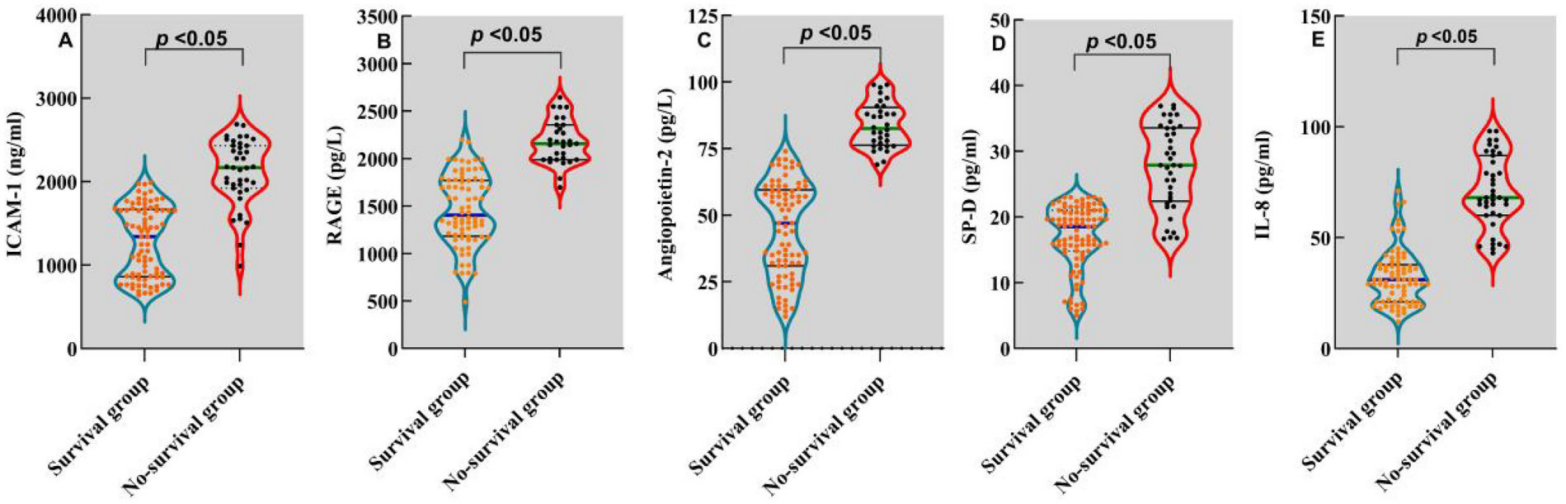
Distribution of risk factors for the prognosis of PARDS. Relative levels of ICAM-1 **(A)**, RAGE **(B)**, Ang-2 **(C)**, SP-D **(D)** and IL-8 **(E)** in children with ARDS of the survival group and non-survival group.

The univariate logistic regression analysis revealed that vWF, IL-8, RAGE, Ang-2, ICAM-1 and SP-D were significantly associated with the mortality of PARDS (all *OR* > 1 and *P* < 0.05). The multivariate logistic regression analysis further demonstrated that IL-8, RAGE, Ang-2, ICAM-1 and SP-D were independent risk factors for the prognosis of PARDS (all *OR* > 1 and *P* < 0.05, Table 2). Compared with those of survival group, children with ARDS in the non-survival group presented significantly higher levels of ICAM-1 (1,438 ± 622 ng/mL vs. 2,126 ± 421 ng/mL, Figure 2A), RAGE (1,498 ± 322 pg/L vs. 2,131 ± 224 pg/L, Figure 2B), Ang-2 (8 ± 20 pg/L vs. 77 ± 21 pg/L, Figure 2C), SP-D (19 ± 6 pg/mL vs. 29 ± 10 pg/mL, Figure 2D), IL-8 (32 ± 16 pg/mL vs. 72 ± 21 pg/mL, *P* < 0.05; Figure 2E).

**Table 2:** Logistic regression analysis of risk factors for the prognosis of PARDS.

	Univariate logistic		Multivariate logistic	
	Adjusted *OR* (95% *CI*)	*P* value	Adjusted *OR* (95% *CI*)	*P* value
D-dimer (×10^9^/L)	2.67 (1.51, 3.56)	0.011	1.62 (1.33, 1.79)	0.053
vWF (mg/L)	2.34 (1.18, 3.46)	0.000	1.64 (0.91, 2.71)	0.056
Blood cell analysis				
Platelet count (×10^9^/L)	0.54 (0.32, 0.98)	0.052		
Leukocyte count (×10^9^/L)	1.03 (0.67, 1.29)	0.318		
Neutrophils (%)	0.98 (0.45, 1.05)	0.334		
Lymphocytes (%)	1.11 (0.76, 1.35)	0.246		
Hb (×10^9^/L)	0.95 (0.76, 1.05)	0.316		
Inflammatory factors				
CRP (mg/L)	1.13 (0.98, 1.19)	0.221		
PCT (mg/L)	1.32 (0.87, 1.81)	0.312		
IL-8 (pg/mL)	2.78 (1.49, 4.12)	0.000	2.12 (1.34, 3.11)	0.023
TNF-α (pg/mL)	1.14 (0.96, 1.59)	0.113		
RAGE (pg/L)	2.69 (1.54, 3.95)	0.000	1.55 (1.21, 2.34)	0.036
Ang-2 (pg/L)	3.01 (1.94, 3.89)	0.000	1.98 (1.49, 2.63)	0.039
ICAM-1 (ng/mL)	2.67 (1.95, 3.03)	0.002	1.35 (1.04, 2.01)	0.044
SP-D (pg/mL)	2.11 (1.43, 3.01)	0.014	1.49 (1.14, 2.43)	0.041

vWF, von Willebrand factor; Hb, hemoglobin; CRP, C-reactive protein; PCT, procalcitonin; IL-8, interleukin 8; TNF-α, tumor necrosis factor alpha; RAGE, receptor for advanced glycation endproducts; Ang-2, angiopoietin-2; ICAM-1, intracellular adhesion molecule-1; SP-D, surfactant protein D.

### 3.4 Predictive potential in mortality of ICAM-2, RAGE, Ang-2 and SP-D in PARDS

**Figure 3:**
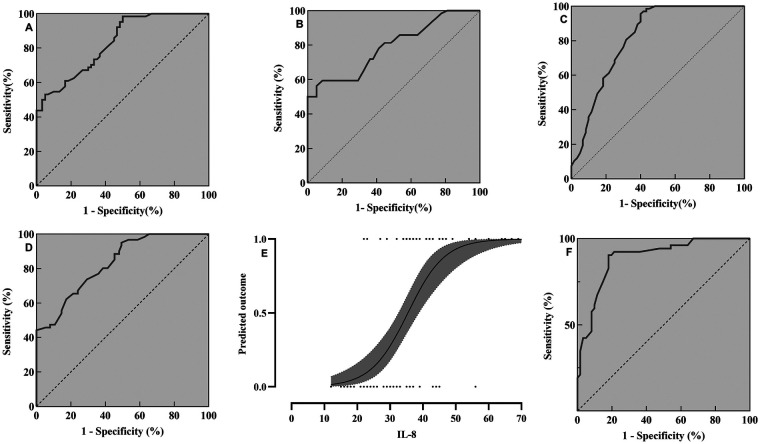
ROC curves of ICAM-1 **(A)**, RAGE **(B)**, Ang-2 **(C)**, SP-D **(D)** and IL-8 in regressive prognosis **(E)** and prediction **(F)** in PARDS.

ROC curves were plotted to illustrate the predictive potential of the above risk factors in discriminating PARDS. The area under the curve (AUC) of ICAM-1 (sensitivity = 75.45%, specificity = 73.34%, Figure 3A), RAGE (sensitivity = 81.21%, specificity = 76.15%, Figure 3B), Ang-2 (sensitivity = 76.54%, specificity = 75.45%, Figure 3C) and SP-D (sensitivity = 72.12%, specificity = 73.41%, Figure 3D) in distinguishing PARDS was 0.754, 0.778, 0.787 and 0.732, respectively. All of them presented acceptable performances in diagnosing PARDS. They demonstrated a strong predictive performance in assessing the mortality of PARDS.

### 3.5 IL-8 is a prognostic factor for PARDS

A significantly higher level of IL-8 was detected in children with ARDS of the non-survival group than survival group. Notably, the AUC of IL-8 in discriminating PARDS was up to 0.898, with the cut-off value of 61.7 pg/mL, sensitivity of 88.12% and specificity of 83.45% (Figures 3E,F).

### 3.6 Correlation of IL-8 with the risk factors of mortality for PARDS

**Figure 4:**
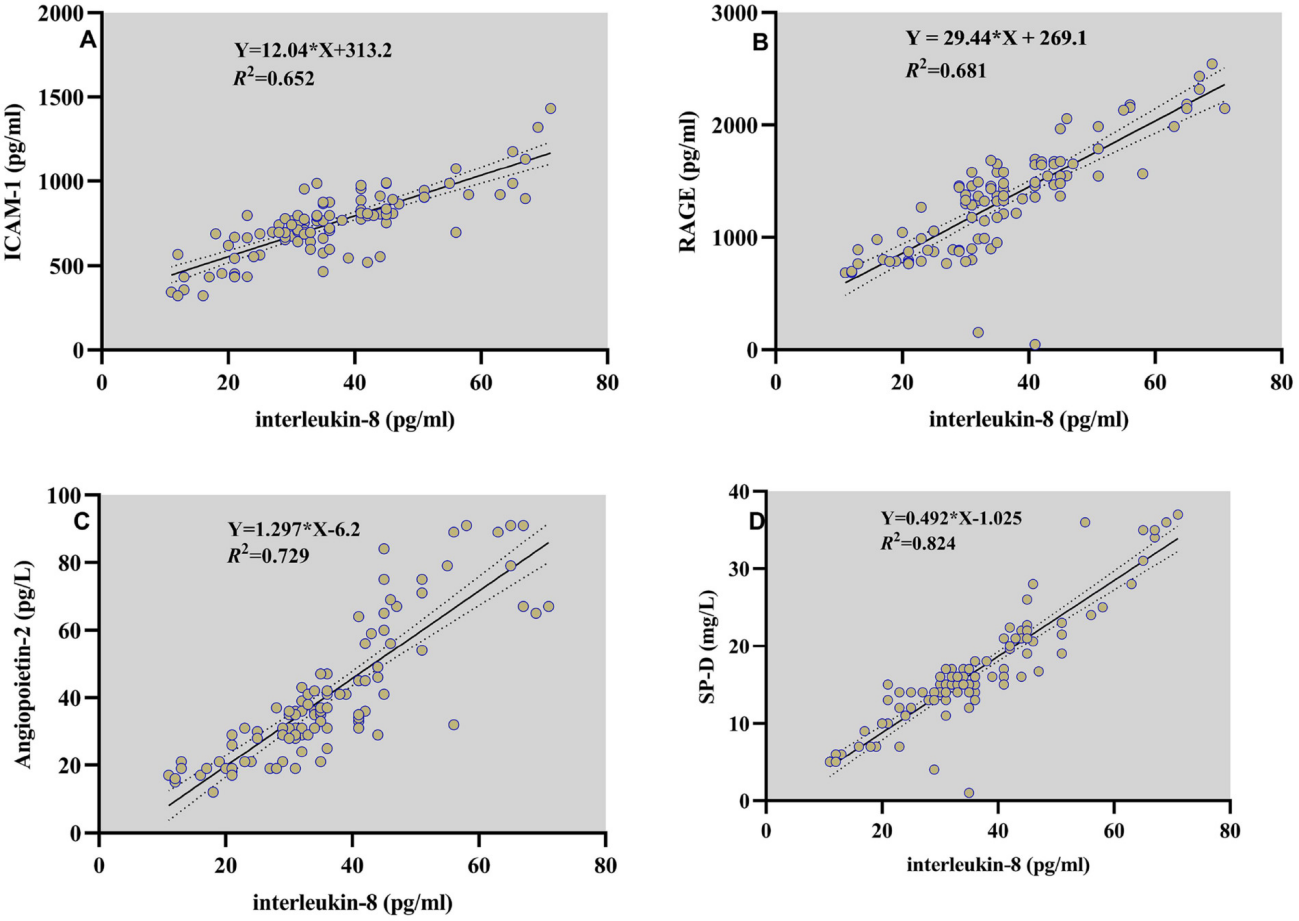
Correlation of IL-8 with ICAM-1 **(A)**, RAGE **(B)**, Ang-2 **(C)** and SP-D **(D)** in children with ARDS.

**Figure 5:**
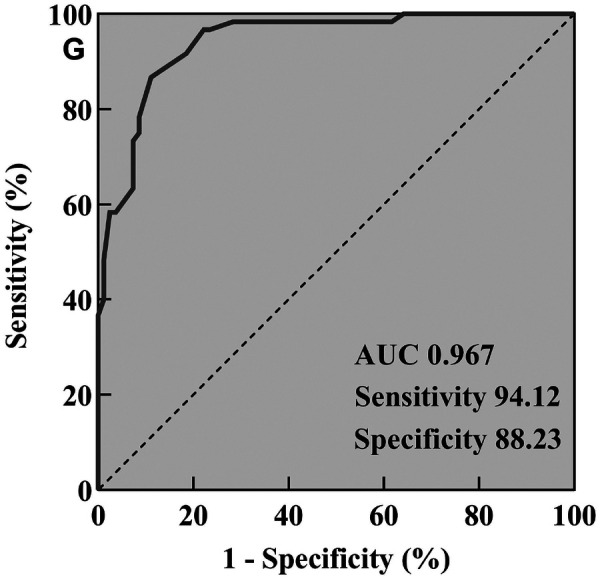
ROC curves of IL-8 combined with ICAM-1, RAGE, Ang-2 and SP-D in distinguishing PARDS.

IL-8 levels in children with ARDS were positively correlated with ICAM-1 (*R*^2^ = 0.652, *P* = 0.035, Figure 4A), RAGE (*R*^2^ = 0.681, *P* = 0.029, Figure 4B), Ang-2 (*R*^2^ = 0.729, *P* = 0.022, Figure 4C) and SP-D (*R*^2^ = 0.824, *P* = 0.018, Figure 4D). Considering the importance of IL-8 and the identified risk factors in PARDS, we investigated the potential of their combination in predicting mortality of PARDS. The AUC of IL-8 combined with ICAM-1, RAGE, Ang-2 and SP-D in predicting mortality was as high as 0.967, with the sensitivity of 94.12% and specificity of 88.23% (Figure 5).

## 4 Discussion

For more than half a century, ARDS has still been blamed for its relatively high morbidity and mortality rates, posing a great threat to the health of children and adults (15–17). The PALICC-2 guidelines offer pediatricians with more objective and effective efforts for diagnosing and treating ARDS; moreover, ongoing research is paying an increasing attention on the impact of ARDS phenotypes on patient prognosis. At present, inflammatory phenotypes have been highlighted for their significance in adults and children with lung diseases. The correlation of the hyperinflammatory state with the prognosis of ARDS has been growingly concerned, especially since after the global epidemic of the COVID-19 (18, 19).

In direct ARDS, acute damages to the alveolar epithelium and capillary endothelium results in the disruption of the endothelial barrier, fluid leakage and pulmonary oedema (20–22). During the pathological process of ARDS, pro-inflammatory factors are responsible for activating platelets and cytokines, and recruiting harmful substances within lung tissues (23). A hyperinflammatory state is typically characterized by severe and uncontrolled inflammatory response involving multiple organs and systems. In comparison to the hypoinflammatory state, the hyperinflammatory phenotype of ARDS usually links with more severe inflammatory responses and worse prognosis.

ICAM-1 is an established regulator of inflammation and vascular injury via mediating endothelial and epithelial barrier function. It is significantly correlated with the severity of pulmonary exudation, oxygenation dysfunction and 28-day mortality in adult ARDS (24–26). Ang-2 is an endogenous ligand for the tyrosine kinase receptor expressed on the surface of endothelial cells, acting as a trigger of increased permeability of endothelial cells (27). Serum Ang-2 is found significantly higher in patients with severe sepsis than those with a milder condition, which is also positively correlated with arterial hypoxemia. Bhandari et al. (28) detected higher Ang-2 levels in plasma and pulmonary edema fluid of ARDS patients than those with hydrostatic pulmonary edema. RAGE is a receptor for transmembrane immunoglobulins and expressed on the basal surface of alveolar type I (ATI) cells (29). It is usually overexpressed in ARDS models, presenting a correlation with the severity of hypoxia (12, 30–32). The damage to alveolar endothelium impairs a normal clearance of alveolar exudate materials, and their accumulation eventually induces pulmonary edema and increases alveolar surface tension (12, 33). SP-D level increases in response to alveolar cell damages. Overexpression of SP-D not only causes oxygenation dysfunction and ventilator-induced lung injury, but also increases the mortality (34–36). In the present study, RAGE, Ang-2, ICAM-1 and SP-D were independent risk factors for the mortality of PARDS, which were significantly higher in non-survived children with ARDS than those of survivors. Our data confirmed that alveolar damage worsened the prognosis of ARDS.

Previous data have confirmed that neutrophils are recruited, migrated, and activated in lung tissue, playing a pivotal role in lung injury. In this process, IL-8 plays a crucial role in transmitting inflammatory signals and activating inflammation, thereby further enhancing the recruitment of inflammatory cells to the affected tissue and exacerbating associated damage (37–39). Overexpression of IL-8 is found as a vital biomarker for predicting a poor prognosis in PARDS. Moreover, upregulated IL-8 exacerbates oxygenation impairment and prolongs the duration of ventilator therapy in severe PARDS (39). Overexpressed IL-8 is considered to cause a hyperinflammatory state, leading to the damages to alveolar epithelial and endothelial cells. Meanwhile, severe pulmonary inflammatory exudation is noted (37, 39–41).

Here, a significant difference was detected in IL-8 levels among children with mild, moderate and severe ARDS. Its level was significantly higher in non-survival group than the survival group. Moreover, IL-8 was positively correlated with RAGE, Ang-2, ICAM-1 and SP-D levels in PARDS. Compared with a single detection, a combination detection of IL-8 with RAGE, Ang-2, ICAM-1 and SP-D presented an extraordinary performance in predicting the mortality of PARDS. In the future, non-invasive measurements of IL-8, RAGE, Ang-2, ICAM-1 and SP-D are believed as a promising approach to identify high-risk population of PARDS.

Several limitations should be concerned. First of all, this was a retrospective study conducted in two pediatric hospitals in northwest China. Totally, 16 patients were excluded due to incomplete key data, which may have influenced the study outcomes. This limitation should be objectively acknowledged as a potential source of bias in the results. Our research needs to be further clinically validated in PARDS patients in other regions. Second, the potential mechanisms underlying the role of IL-8 in ARDS remain unclear. Third, clinical benefits of anti-IL-8 autoantibodies to PARDS are the future spotlight to be deeply mined. In future research on PARDS, prospective cohort studies should be implemented to rigorously address the heterogeneity associated with PARDS and to generate more robust scientific evidence for informing clinical diagnosis and treatment strategies.

## 5 Conclusion

IL-8 is overexpressed in children with ARDS, showing a prognostic potential particularly in combination with RAGE, Ang-2, ICAM-1 and SP-D in PARDS.

## Data Availability

The original contributions presented in the study are included in the article/Supplementary Material, further inquiries can be directed to the corresponding author.
